# Supplementary material for: Causal Relationship between Aging and Anorexia Nervosa: A White-Matter-Microstructure-Mediated Mendelian Randomization Analysis
Source: Biomedicines. 2024 Aug 16;12(8):1874. doi: 10.3390/biomedicines12081874 (PMC11351342; doi:10.3390/biomedicines12081874)
Supplement: Supplementary file 1 [file biomedicines-12-01874-s001.zip › Supplementary Figure/Figure S8/Leaveoneout plot of MR analysis for TL to ubm-b-1484.pdf]

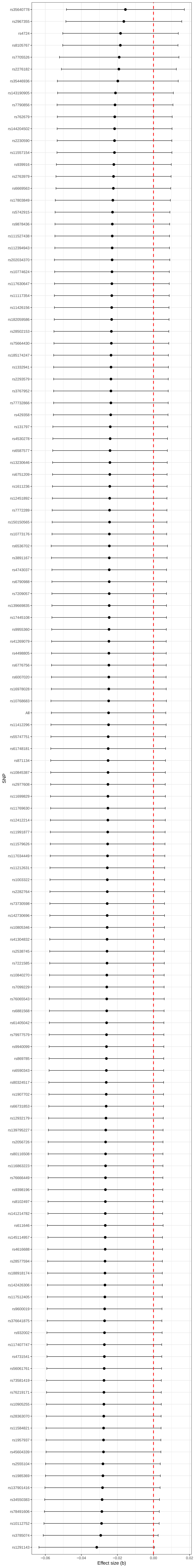
rs35640778
rs2967355
rs4724
rs8105767
rs7705526
rs2276182
rs35446936
rs143190905
rs7790856
rs762679
rs144204502
rs2230590
rs11557154
rs939916
rs2763979
rs6669563
rs17803849
rs5742915
rs9878436
rs111527438
rs112394943
rs202034370
rs10774624
rs117630647
rs11117354
rs11426156
rs182059586
rs28502153
rs75664430
rs185174247
rs1332941
rs2293579
rs3767952
rs77732866
rs429358
rs131797
rs4530278
rs6587577
rs13230646
rs6751209
rs1611236
rs12451892
rs7772289
rs150150565
rs10773176
rs6536702
rs3891167
rs4743037
rs6790988
rs7209057
rs139669835
rs17445108
rs9955360
rs41269079
rs4498805
rs6776756
rs6007020
rs16978028
rs10768683
All
rs11412296
rs55747751
rs61748181
rs871134
rs10845387
rs2977608
rs11699829
rs11769630
rs12412214
rs11991877
rs11579626
rs117034449
rs11212631
rs1003322
rs2282764
rs73730598
rs142730696
rs10805346
rs41304832
rs2538745
rs7221585
rs10840270
rs7099229
rs76065543
rs6881568
rs61405042
rs79977579
rs9940099
rs869785
rs6590343
rs80324517
rs1907702
rs66731853
rs12932179
rs139795227
rs2056726
rs80116508
rs116863223
rs76666449
rs9398196
rs8102497
rs141214782
rs611646
rs145114957
rs4616688
rs28577594
rs188918174
rs142426306
rs117512405
rs9600019
rs376641875
rs932002
rs117407747
rs4731541
rs56061761
rs73581419
rs76219171
rs10905255
rs28363070
rs11584821
rs1957937
rs45604339
rs2555104
rs1985369
rs137901416
rs34550383
rs78491606
rs10112752
rs3785074
rs1291143
SNP
−0.06
−0.04
−0.02
0.00
0.02
Effect size (b)
